# Supplementary material for: Identification of Freezing-Responsive microRNAs and Their Targets in Chinese Jujube by Small RNA and Degradome Sequencing
Source: Int J Mol Sci. 2026 Sep 4;27(17):7912. doi: 10.3390/ijms27177912 (PMC13566890; doi:10.3390/ijms27177912)
Supplement: Supplementary file 1 [file ijms-27-07912-s001.zip › Supplementary Material S2/novel_miR_22.pdf]

Provisional ID : novel_miR_22
Score total : 0.4
Score for star read(s) : -1.3
Score for read counts : 0
Score for mfe : 0.1
Score for randfold : 1.6
Score for cons. seed :
Total read count : 56
Mature read count : 56
Loop read count : 0
Star read count : 0

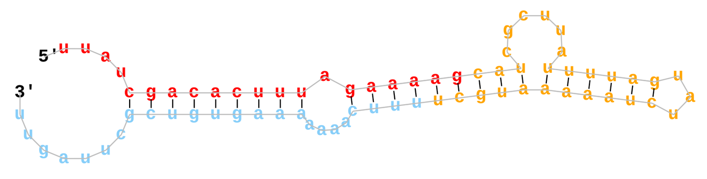

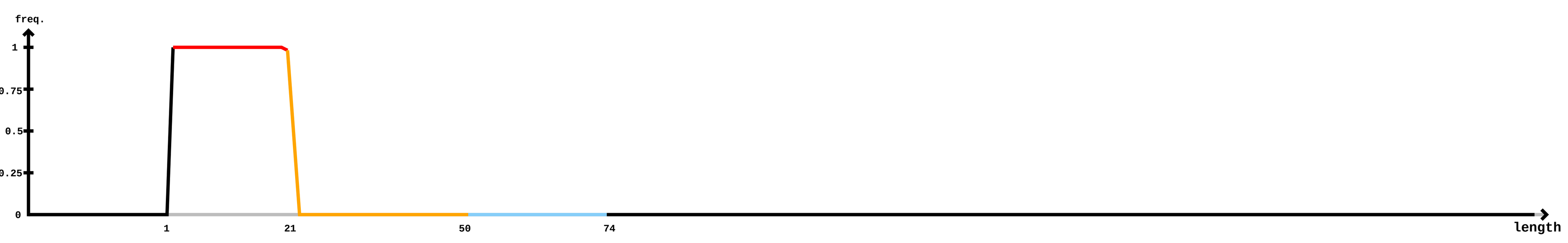


```
                     Mature                        Star
5'- gaaaaguauauuguccuaauauuuaucgacacuuuagaaaagcaucgcuuauuuuaguaucuaaaaaugcuuuucaaaaaaagugucgcuuaguuacauauuuagugugcuuuuauacaaaaagcguugccuuuuguuuuguuucauuuuuuuuuuuuucaauuugaaaguugcuuuuagcuuucugaaauuaugauuguuuaauagcuaaauucaugcaaaauaauuaauagacaacaacguucaaaua -3'  exp
    .((((((((((((........(((.(((((((((.((((((((((.....((((((...))))))))))))))))....)))))))))..))).........)))))))))))........((((((....((((((................(((((....((((((((....))))))))))))))...(((((((((....((.........)).)))))))))..))))))))))))......  reads  mm  sample
    .....................uuaucgacacuuuagaaaU.......................................................................................................................................................................  1  1  LT-3
    .....................uuaucgacacuuuNgaaaag......................................................................................................................................................................  1  1  LCK-1
    .....................uuaucgacacuuuagaaaag......................................................................................................................................................................  26  0  LCK-1
    .....................uuaucgacacuuuagaaaag......................................................................................................................................................................  27  0  LCK-2
    .....................uuaucgCcacuuuagaaaag......................................................................................................................................................................  1  1  LCK-2
```
